# Supplementary material for: Adipocyte HIF2α functions as a thermostat via PKA Cα regulation in beige adipocytes
Source: Nat Commun. 2022 Jun 7;13:3268. doi: 10.1038/s41467-022-30925-0 (PMC9174489; doi:10.1038/s41467-022-30925-0)
Supplement: Supplementary file 1 — Supplementary Information [file 41467_2022_30925_MOESM1_ESM.pdf]

Supplementary Information

Adipocyte HIF2α functions as a thermostat via PKA Cα regulation in beige adipocytes

Han et al.

Supplementary Fig. 1

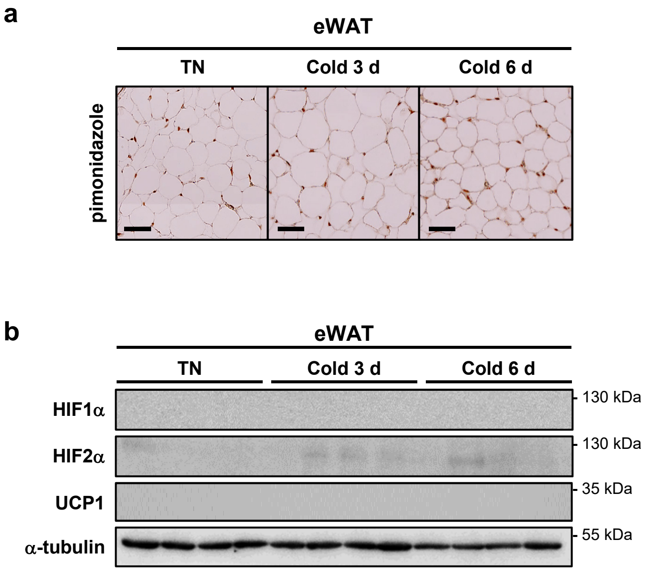

Supplementary Fig. 1 Hypoxia-related phenotypes in eWAT upon cold.

a Immunohistochemical analysis of eWAT section upon TN or cold exposure using anti-pimonidazole antibody and DAB staining. Scale bars, 50 μm. **b** Western blot analysis of HIFα and UCP1 in eWAT upon TN or cold exposure.

Supplementary Fig. 2

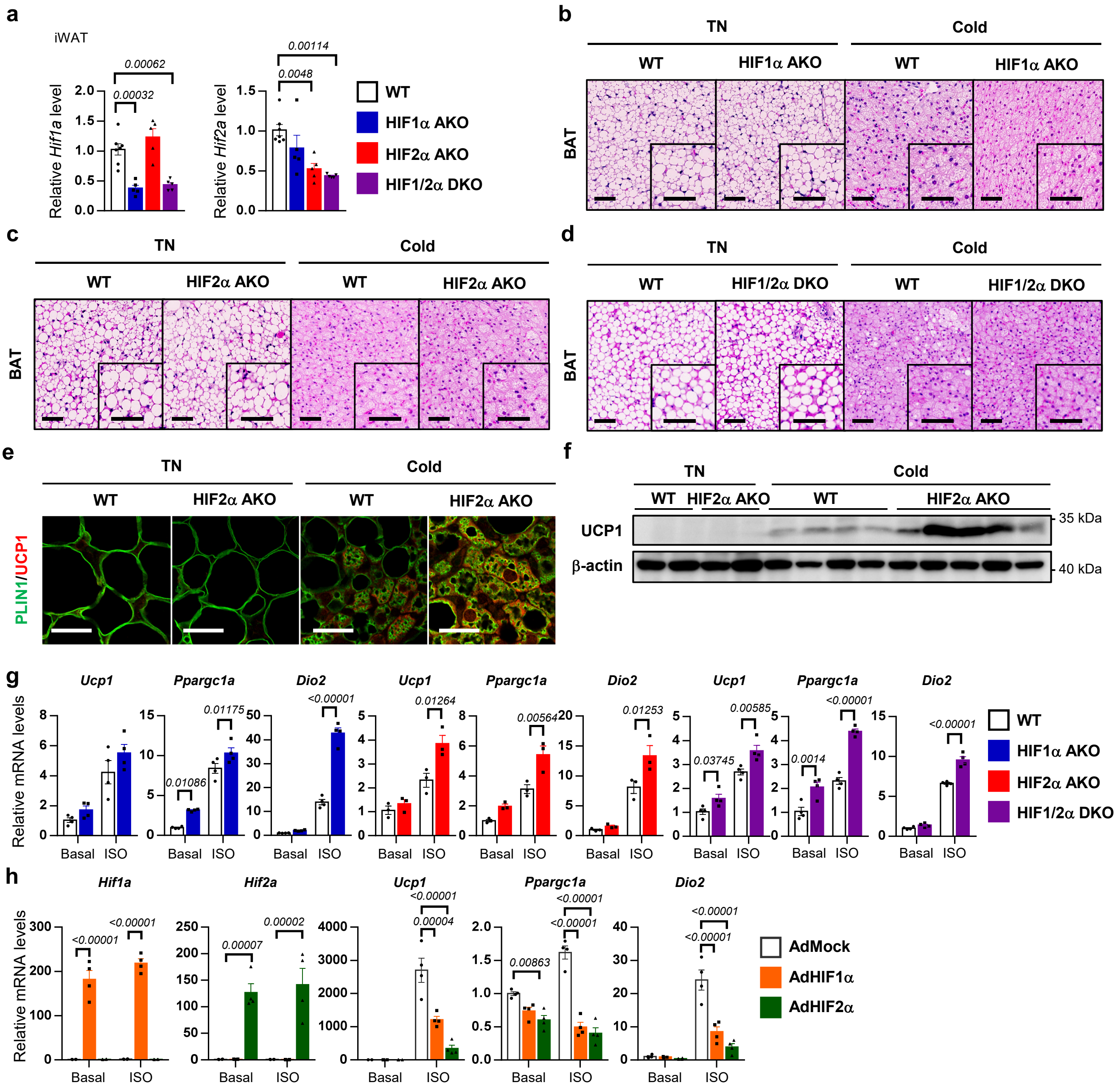

Supplementary Fig. 2 Characterization of HIFα deficiency in adipocytes.

a mRNA levels in iWAT from WT (n=7), HIF1α AKO (n=5), HIF2α AKO (n=5), and HIF1/2α DKO (n=5) mice. **b-d** Representative images of H&E staining of BAT from WT, (**b**) HIF1α AKO, (**c**) HIF2α AKO, and (**d**) HIF1/2α DKO mice upon TN or cold exposure (3 d). Scale bars, 50 μm. **e** Representative immunofluorescence images of iWAT sections of WT and HIF2α AKO mice upon cold exposure (3 d). Scale bars, 25 μm. **f** Western blot analysis of UCP1 from iWAT of WT and HIF2α AKO mice upon TN or cold exposure (3 d). **g** mRNA levels in beige adipocytes from WT (left panels; n=4, middle panels; n=3, right panels; n=4), HIF1α AKO (n=4), HIF2α AKO (n=3), and HIF1/2α DKO (n=4) mice without or with ISO (5 μM, 4 h). **h** The mRNA levels in beige adipocytes (n=4) infected with Admock, AdHIF1α, or AdHIF2α and without or with ISO (5 μM, 4 h). Data are expressed as the mean ± SEM by one-way ANOVA in (**a**) or two-way ANOVA in (**g**,**h**) followed by Holm-Sidak's multiple comparisons test. ISO, isoproterenol

Supplementary Fig. 3

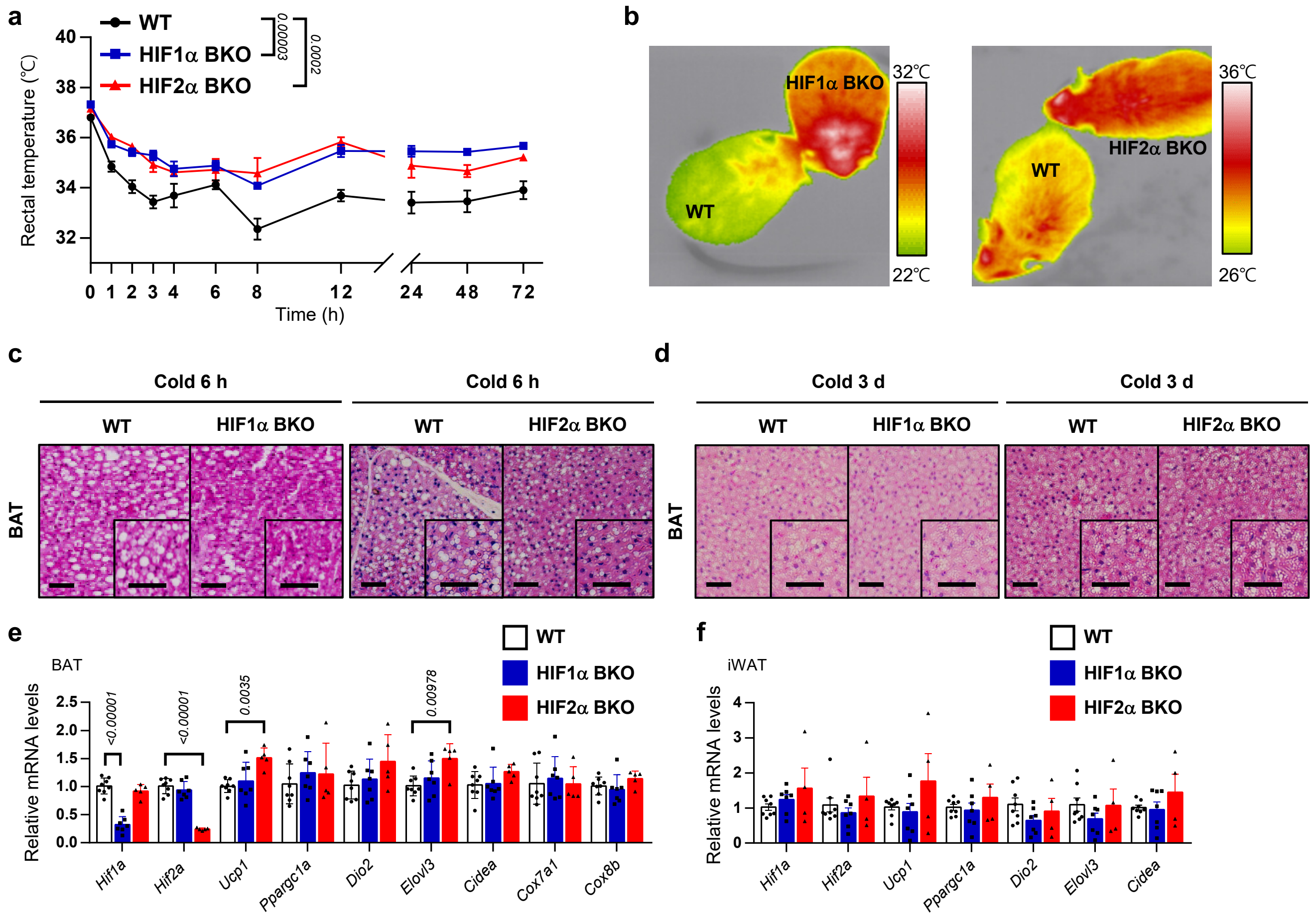

Supplementary Fig. 3 Characterization of BAT in HIFα deletion models.

a Rectal temperature of WT (n=8), HIF1α BKO (n=10), and HIF2α BKO (n=8) mice during cold exposure. **b** Infrared images of body surface temperature of WT, HIF1α BKO, and HIF2α BKO upon cold exposure (4 h). **c**, **d** Representative images of H&E staining of BAT from WT, HIF1α BKO, and HIF2α BKO upon (**c**) 6 hours and (**d**) 3 days of cold exposure. Scale bars, 50 μm. **e** mRNA levels in BAT from WT (n=8), HIF1α BKO (n=7), and HIF2α BKO (n=5) upon cold exposure (3 d). **f** mRNA levels in BAT from WT (n=8), HIF1α BKO (n=7), and HIF2α BKO (n=4) upon cold exposure (3 d). Data are expressed as the mean ± SEM by one-way ANOVA in (**e**,**f**) or two-way repeated-measures ANOVA in (**a**) followed by Holm-Sidak's multiple comparisons test.

Supplementary Fig. 4

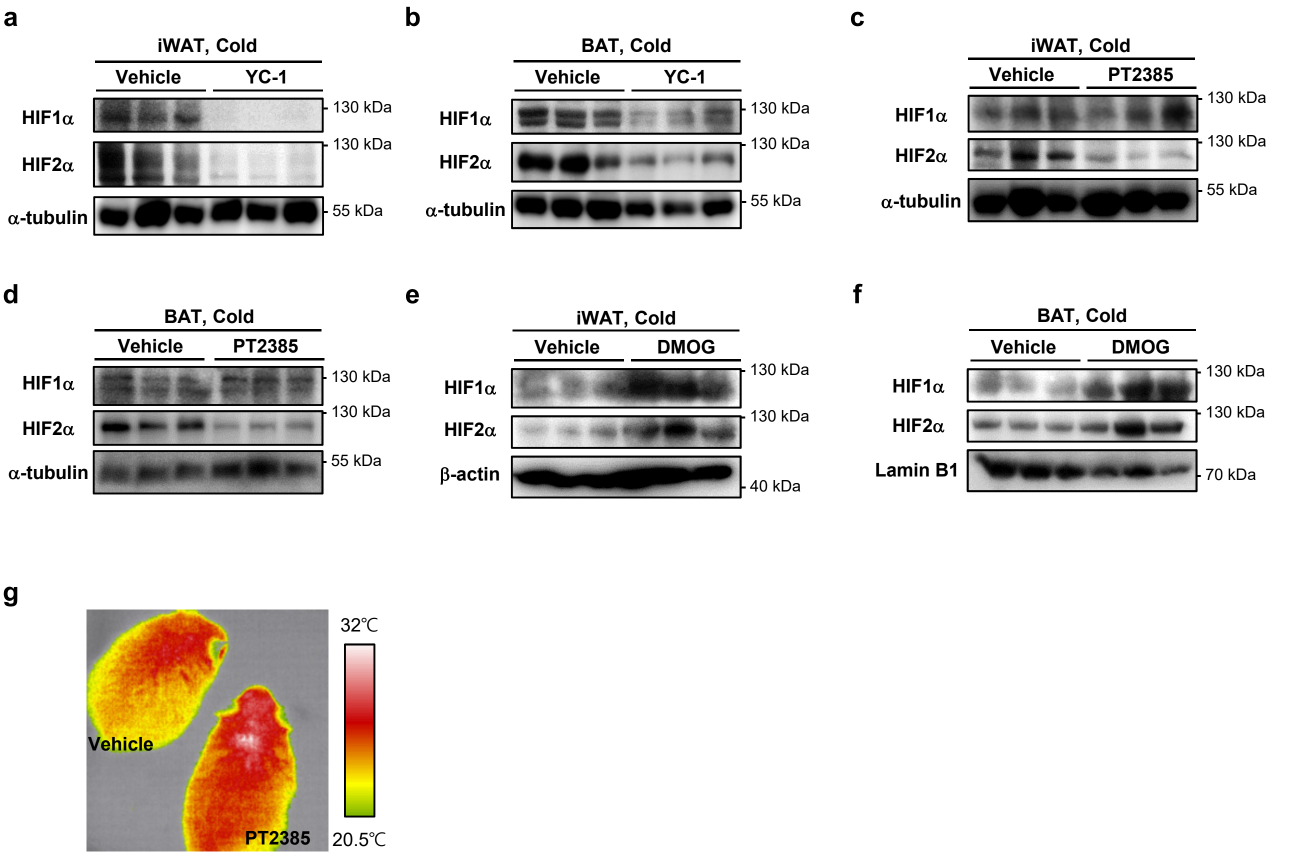

Supplementary Fig. 4 Effects of pharmacological HIFα modulators on mice.

a, b Western blot analysis of HIFα in (**a**) iWAT or (**b**) BAT from vehicle- and YC-1 (30 mg/kg, daily)-administered mice upon cold exposure (3 d). **c, d** Western blot analysis of HIFα in (**c**) iWAT or (**d**) BAT from vehicle- and PT2385 (10 mg/kg, twice a day)-administered mice upon cold exposure (3 d). **e, f** Western blot analysis of HIFα in (**e**) iWAT or (**f**) BAT from vehicle- and DMOG (40 mg/kg, daily)-administered mice upon cold exposure (3 d). **g** Infrared images of body surface temperature of vehicle- and PT2385-administered mice upon cold exposure (4 h).

Supplementary Fig. 5

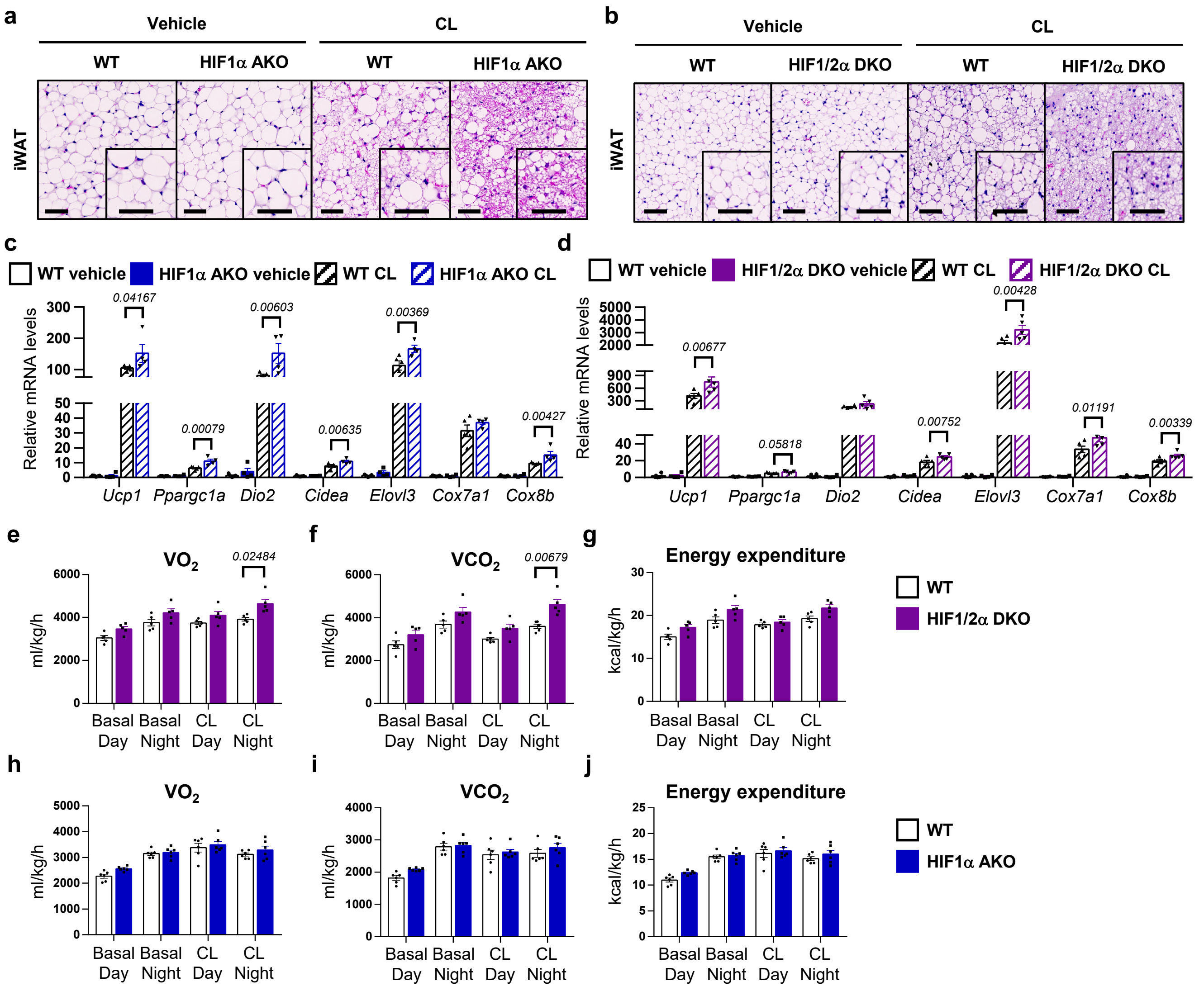

Supplementary Fig. 5 Phenotypes of HIF1α AKO and HIF1/2α DKO upon CL administration.

a, **b** Representative images of H&E staining of iWAT from WT, (**a**) HIF1α AKO, and (**b**) HIF1/2α DKO mice upon daily CL administration (0.5 mg/kg, 4 d). Scale bars, 50 μm. **c**, **d** The mRNA levels in iWAT from (**c**) WT (n=5) and HIF1α AKO (n=4), and (**d**) WT (vehicle; n=6, CL; n=4) and HIF1/2α DKO (vehicle; n=4, CL; n=5) mice upon daily CL administration (0.5 mg/kg, 4 d). **e-j** VO_2, VCO_2, and energy expenditure for (**e**,**f**,**g**) WT (n=5) and HIF1/2α DKO (n=5), and (**h**,**i**,**j**) WT (n=6) and HIF1α AKO (n=6) mice. Data are expressed as the mean ± SEM by two-way ANOVA followed by Holm-Sidak's multiple comparisons test.

Supplementary Fig. 6

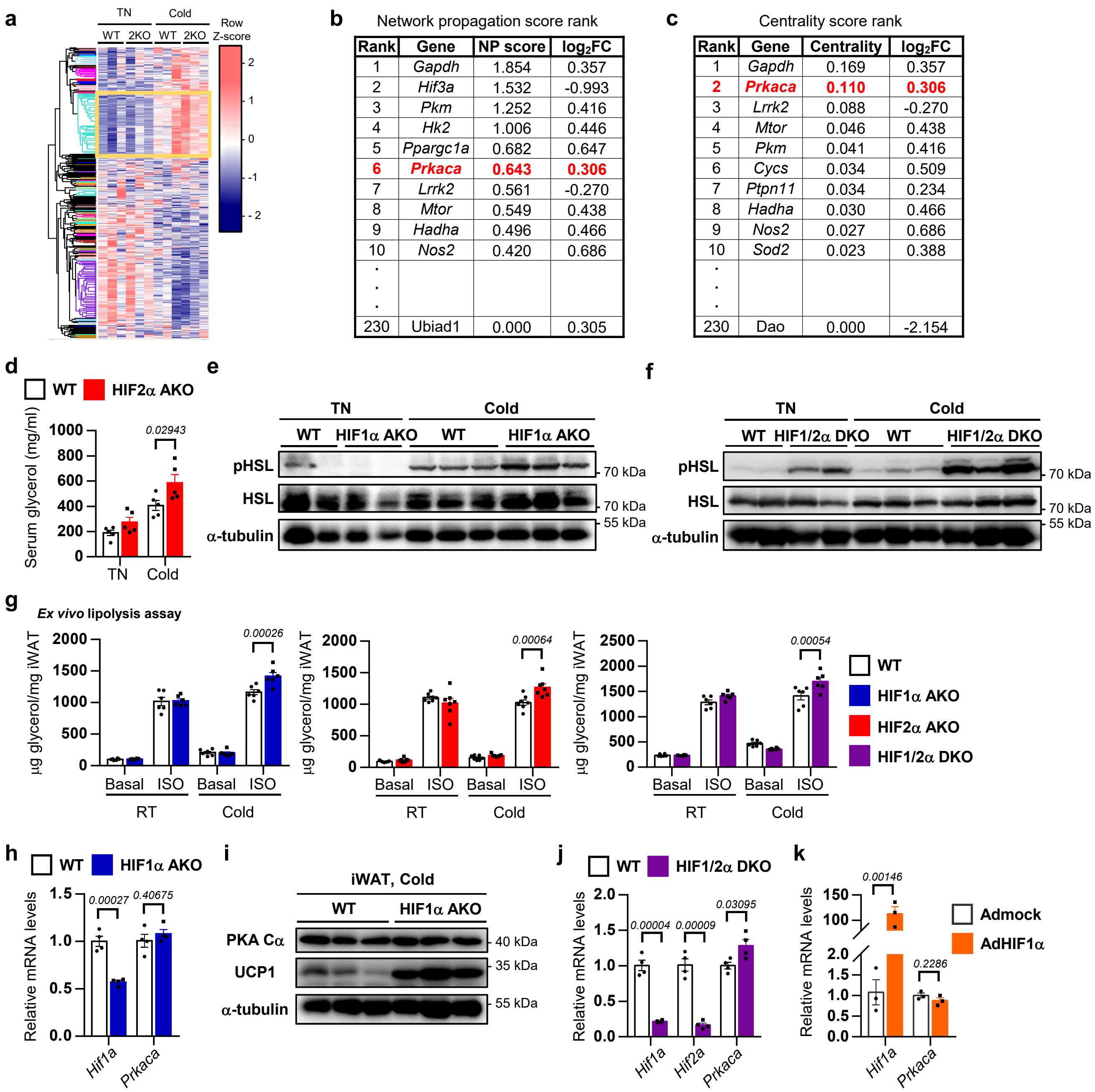

Supplementary Fig. 6 Regulation of *Prkaca* and PKA signaling pathway by HIF2α.

a Heatmap showing gene expression patterns. **b**, **c** (**b**) NP score ranks and (**c**) Betweenness centrality ranks of thermogenesis-related genes in the condition-specific network. **d** Serum glycerol concentration of WT (n=5) and HIF2α AKO (n=5) mice upon TN or cold exposure (6 h). **e**, **f** Western blot analysis of PKA signaling in iWAT from WT, (**e**) HIF1α AKO, and (**f**) HIF1/2α DKO mice upon TN or cold exposure (6 h). **g** Glycerol concentration in culture media of iWAT explants from WT (left panel; n=6, middle panel; n=7, right panel; n=6), HIF1α AKO (n=6), HIF2α AKO (n=7), and HIF1/2α DKO (n=6) mice RT and cold exposed (3 d) mice without or with ISO (5 μM, 3 h). **h** mRNA levels in beige adipocytes from WT (n=4) and HIF1α AKO (n=4) mice. **i** Western blot analysis of PKA Cα and UCP1 in iWAT from WT and HIF1α AKO mice upon cold exposure (3 d). **j** mRNA levels in beige adipocytes from WT (n=4) and HIF1/2α DKO (n=4) mice. **k** mRNA levels in beige adipocytes infected with Admock (n=3) or AdHIF1α (n=3). Data are expressed as the mean ± SEM by two-tailed unpaired Student t tests in (**h**,**j**,**k**) or two-way ANOVA in (**d**,**g**) followed by Holm-Sidak's multiple comparisons test. ISO, isoproterenol

Supplementary Fig. 7

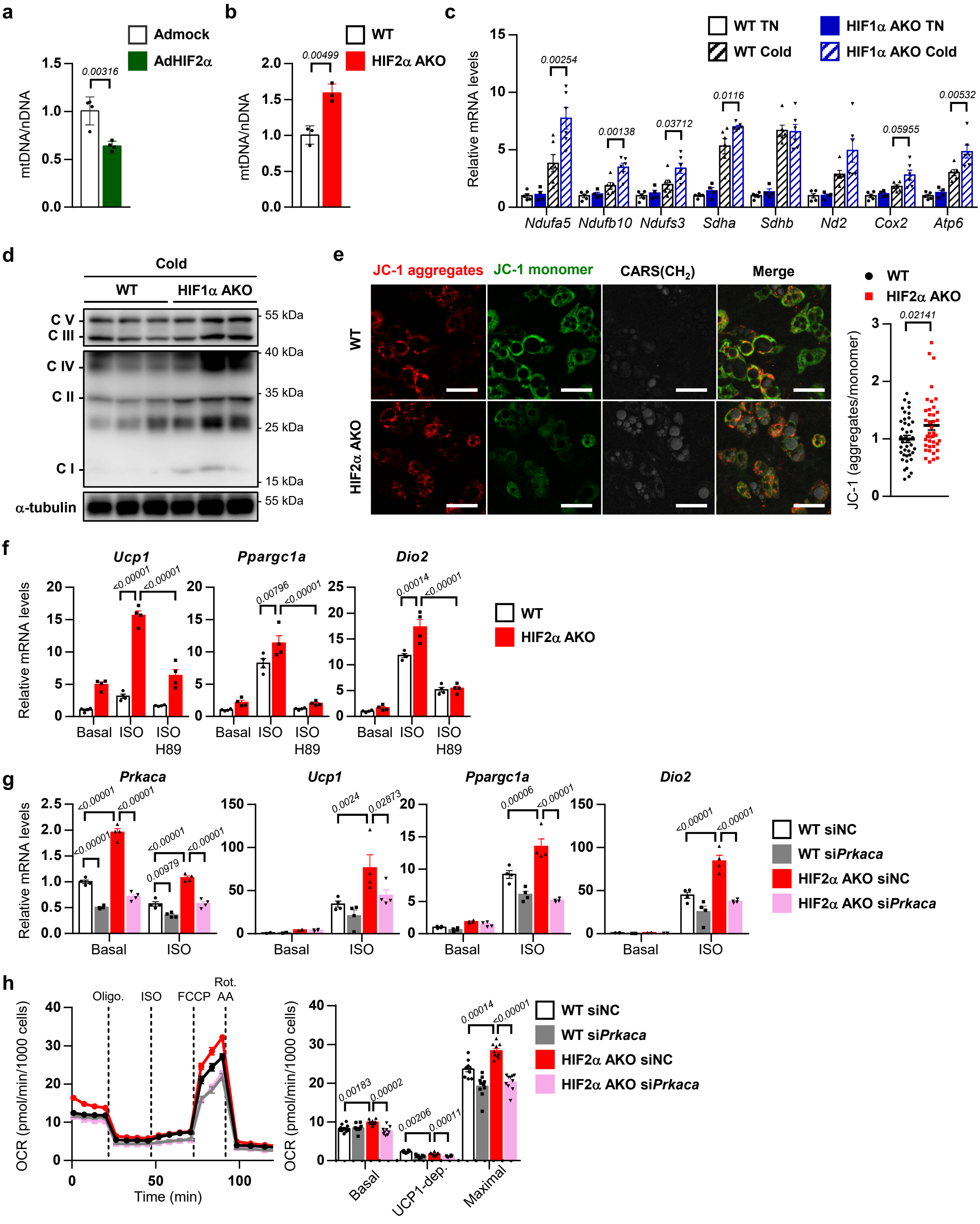

Supplementary Fig. 7 Mitochondrial changes upon HIFα modulation.

a Relative mtDNA contents in beige adipocytes infected with Admock (n=4) or AdHIF2α (n=4). **b** Relative mtDNA contents in beige adipocytes from WT (n=3) and HIF2α AKO (n=3) mice. **c** mRNA levels in iWAT from WT (TN; n=5, Cold; n=6) and HIF1α AKO (TN; n=4, Cold; n=6) mice upon TN or cold exposure (3 d). **d** Western blot analysis of OXPHOS complexes in iWAT from WT and HIF1α AKO mice upon cold exposure (3 d). **e** Representative JC-1 staining images and quantification of the red/green ratio in beige adipocytes (n=40) from WT and HIF2α AKO mice. Scale bars, 25 μm. **f** mRNA levels in beige adipocytes from WT (n=4) and HIF2α AKO (n=4) mice without or with ISO (5 μM, 4 h) upon 1 h pre-incubation of H89 (50 μM). **g** mRNA levels in beige adipocytes from WT (n=4) and HIF2α AKO (n=4) mice transfected with siNC or si*Prkaca* without or with ISO (5 μM, 4 h). **h** OCRs and quantification in beige adipocytes from WT (n=10) and HIF2α AKO (n=10) mice transfected with siNC or si*Prkaca*. Data are expressed as the mean ± SEM by two-tailed unpaired Student t tests in (**a**,**b**,**e**) or two-way ANOVA in (**c**,**f**,**g**,**h**) followed by Holm-Sidak's multiple comparisons test. ISO, isoproterenol

Supplementary Fig. 8

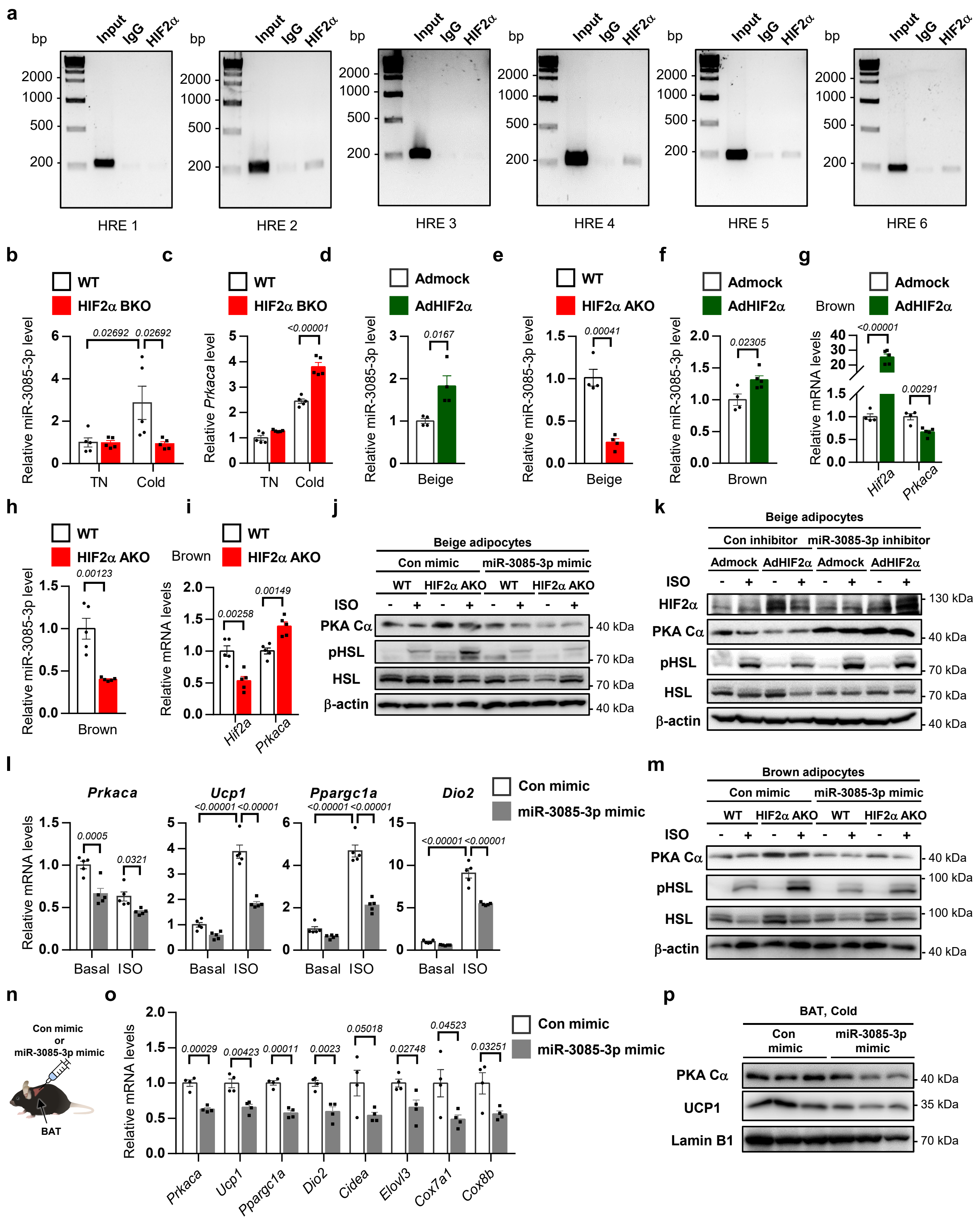

Supplementary Fig. 8 Characterization of miR-3085-3p.

a Images of PCR product from ChIP analysis. **b**, **c** (**b**) miR-3085-3p and (**c**) *Prkaca* level in BAT from WT (n=5) and HIF2α BKO (n=5) mice upon TN or cold exposure (3 d). **d** miR-3085-3p level in beige adipocytes infected with Admock (n=4) or AdHIF2α (n=4). **e**, miR-3085-3p level in beige adipocytes from WT (n=4) and HIF2α AKO (n=4) mice. **f**, **g** (**f**) miR-3085-3p and (**g**) mRNA levels in brown adipocytes infected with Admock (n=4) or AdHIF2α (n=5). **h**, **i** (**h**) miR-3085-3p and (**i**) mRNA levels in beige adipocytes from WT (n=5) and HIF2α AKO (n=5) mice. **j** Western blot analysis of PKA Cα and PKA signaling in beige adipocytes from WT and HIF2α AKO mice transfected with con or miR-3085-3p mimic without or with ISO (5 μM, 1 h). **k** Western blot analysis of beige adipocytes infected with Admock or AdHIF2α upon con or miR-3085-3p inhibitor transfection without or with ISO (5 μM, 1 h). **l** mRNA levels in brown adipocytes transfected with con (n=5) or miR-3085-3p mimic (n=5) without or with ISO (5 μM, 4 h). **m** Western blot analysis of PKA Cα and PKA signaling in brown adipocytes from WT and HIF2α AKO mice transfected with con or miR-3085-3p mimic without or with ISO (5 μM, 1 h). **n** Experimental scheme of miRNA mimic injection. **o** mRNA levels in BAT from mice with con or miR-3085-3p mimic injection upon cold exposure (3 d). **p** Western blot analysis of PKA Cα and UCP1 in BAT from mice with con or miR-3085-3p mimic injection upon cold exposure (3 d). Data are expressed as the mean ± SEM by two-tailed unpaired Student t tests in (**d**-**i**,**o**) or two-way ANOVA in (**b**,**c**,**l**) followed by Holm-Sidak's multiple comparisons test. ISO, isoproterenol

Supplementary Fig. 9

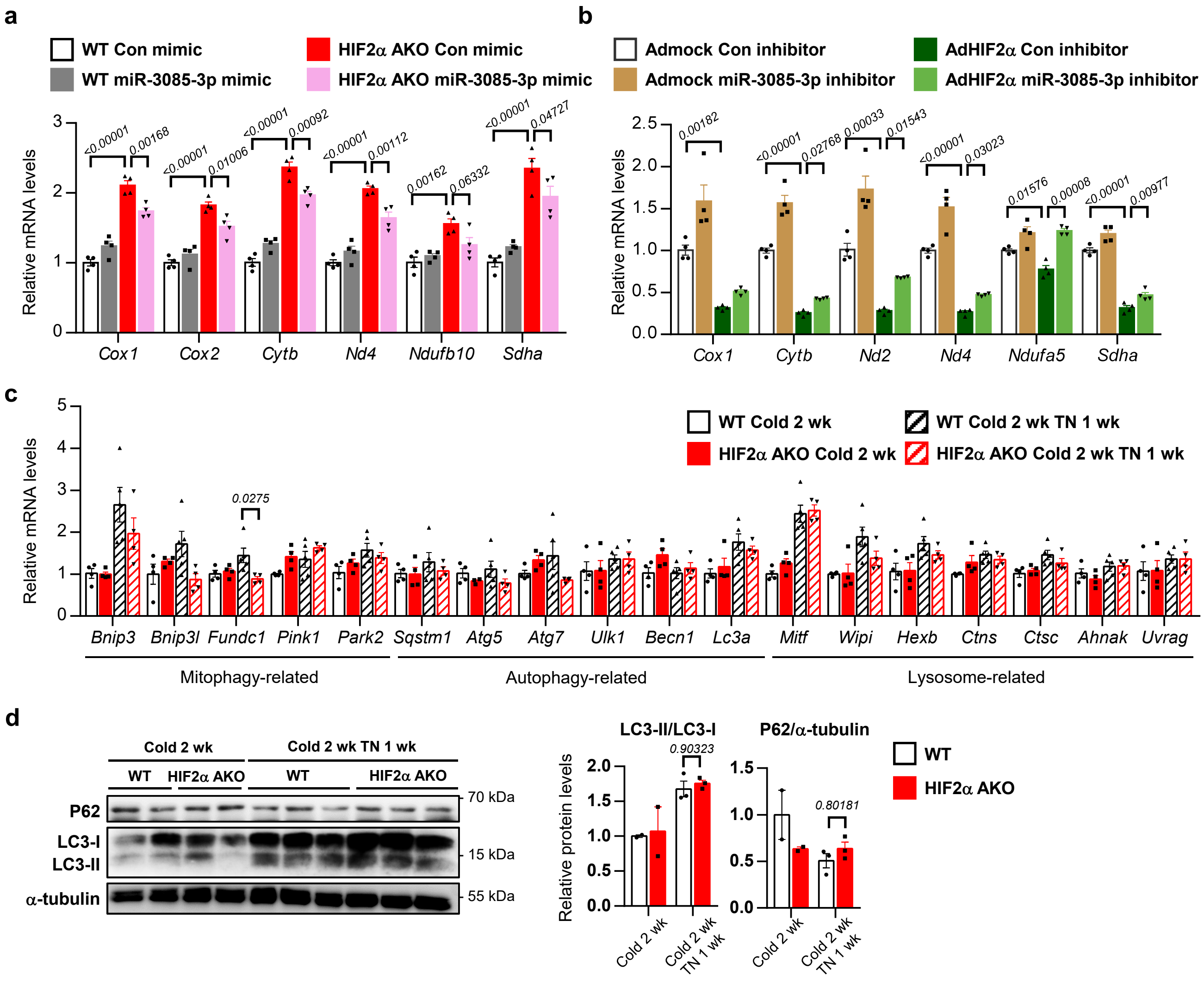

Supplementary Fig. 9 Mitochondrial and autophagy regulation by HIF2α upon re-warming.

a mRNA levels in beige adipocytes from WT (n=4) and HIF2α AKO (n=4) mice transfected with con or miR-3085-3p mimic. **b** mRNA levels in beige adipocytes infected with Admock (n=4) or AdHIF2α (n=4) upon con or miR-3085-3p inhibitor transfection. **c** mRNA levels in iWAT from WT (n=5) and HIF2α AKO (Cold 2 wk; n=4, Cold 2 wk + TN 1 wk; n=5) mice upon cold exposure or re-warming (cold 2 wk + TN 1 wk). **d** Western blot analysis and quantification of autophagy-related proteins in iWAT from WT and HIF2α AKO mice upon cold exposure or re-warming (cold 2 wk + TN 1 wk). Data are expressed as the mean ± SEM by two-way ANOVA followed by Holm-Sidak's multiple comparisons test.

Supplementary Table 1

Gene	Forward	Reverse
**Ucp1**	CTTTGCCTCACTCAGGATTGG	ACTGCCACACCTCCAGTCATT
**Dio2**	CAGTGTGGTGCACGTCTCCAATC	TGAACCAAAGTTGACCACCAG
**Ppargc1a**	CCCTGCCATTGTTAAGACC	TGCTGCTGTTCCTGTTTTC
**Elvol3**	ATGCAACCCTATGACTTCGAG	ACGATGAGCAACAGATAGACG
**Cidea**	GCCGTGTTAAGGAATCTGCTG	TGCTCTTCTGTATCGCCCAGT
**Cox7a1**	CAGCGTCATGGTCAGTCTGT	AGAAAACCGTGTGGCAGAGA
**Cox8b**	GAACCATGAAGCCAACGACT	GCGAAGTTCACAGTGGTTCC
**Cyclophilin**	CAGACGCCACTGTCGCTTT	TGTCTTTGGAACTTTGTCTG
**Hif1a**	TGCTCATCAGTTGCCACTTC	CATGTCGCCGTCATCTGTTA
**Hif2a**	TGAGTTGGCTCATGAGTTGC	TTGCTGATGTTTTCCGACAG
**Prkaca**	CCCGTTCCTGGTCAAACTTG	AGATTCTCGGGCTTCAGGTC
**Prkar1a**	TTACGCCCTTTTGTCGTTTT	AACAGCAACTGTCCCTTTGG
**Prkar2b**	AGGCCAATCTGTATGTTTGGA	CCAATAGGCCAAGGCTAACA
**Ndufa5**	ATCACCTTCGAGAAGCTGGA	ACTTCACCACCCTGAAGCAA
**Ndufb10**	TGGAGCAGTTCACCAAAGTG	TTCCAGCATTCTCTGCTTCT
**Ndufs3**	TTATGGCTTCGAGGGACATC	ATTCTTGTGCCAGCTCCACT
**Sdha**	GGAACACTCCAAAAACAGACCT	CCACCACTGGGTATTGAGTAGAA
**Sdhb**	AATTTGCCATTTACCGATGGGA	AGCATCCAACACCATAGGTCC
**Nd2**	GCCTGGAATTCAGCCTACTAGC	GGCTGTTGCTTGTGTGACGA
**Nd4**	ATAATTATAACTAGCTCAATCTGC	TCGTAGTTGGAGTTTGCTAG
**Nd6**	TGTATGAGGTTGATGATGTTGG	CCGCAAACAAAGATCACCC
**Cox1**	ACACAACTTTCTTTGATCCCG	AGAATCAGAACAGATGCTGG
**Cox2**	ATAATCCCAACAAACGACCT	CTCGGTTATCAACTTCTAGCA
**Cycs**	CCAAATCTCCACGGTCTGTT	GTCTGCCCTTTCTCCCTTCT
**Cytb**	CCTTCATGTCGGACGAGGCTT	TGCTGTGGCTATGACTGCGAA
**Atp6**	TGGCATTAGCAGTCCGGCTT	ATGGTAGCTGTTGGTGGGCT
**Bnip3**	TCCTGGGTAGAACTGCACTTC	GCTGGGCATCCAACAGTATTT
**Bnip3l**	TGTCTCACTTAGTCGAGCCGC	TGGGTAGCTCCACCCAGGAA
**Fundc1**	TTCCGGACCTATGGTAGAAAAA	CCAACCTTCTGGAATAAAAATCC
**Pink1**	CCCACACCCTAACATCATCC	ACTGGGAGTCTGCTCCTCAA
**Park2**	TTCTGACACCAGCATCTTGC	CTTCTCCTCCGTGGTCTCTG
**Sqstm1**	ATGTGGAACATGGAGGGAAGA	GGAGTTCACCTGTAGATGGGT
**atg5**	AGATGGACAGCTGCACACAC	GCTGGGGGACAATGCTAATA
**atg7**	TCCGTTGAAGTCCTCTGCTT	CCACTGAGGTTCACCATCCT
**Ulk1**	CTGACTTTGGATTCGCTCGG	ACTGGTAGACAATGGTGCCA
**Becn1**	TTTCAGACTGGGTCGCTTG	CCATAGGGAACAAGTCGGTAC
**Lc3a**	CATGAGCGAGTTGGTCAAGA	TTGACTCAGAAGCCGAAGGT
**Mitf**	AAGTCGGGGAGGAGTTTCACG	GGAGCTTAACGGAGGCTTGGA
**Wipi**	GCGCTCCGAGGGGAAGTTAT	CCCTTCTGACTTCCACGGCA
**Hexb**	CTCTTTCGGGACTTTCACCA	CCATGGCATCCAGAGTTTTT
**Ctns**	ATGAGGAGGAATTGGCTGCTT	ACGTTGGTTGAACTGCCATTTT
**Ctsc**	TGCCACATCTGAGGAACAAA	CACCAGGACTCCTCTGCATT
**Ahnak**	ACACTGTTGGCTTGAAGTTGC	CTGGGCCATCATGCAGATTGT
**Uvrag**	GACTTTGGAATAATGCCGGATCG	CAGCCCATCCAGGTAGACTTT

mtDNA	Forward	Reverse
**18s rRNA**	CGCGGTTCTATTTTGTTGGT	AGTCGGCATCGTTTATGGTC
**Nd4**	TGCATCAATCATAATCCAAACTCCATGA	GGCAGAATAGGAGTGATGATGTGA

ChIP	Forward	Reverse
**HRE 1**	GGCAGAGTGAGGAAAGCAAA	CCTCAGCCCTTAAGCCTCTT
**HRE 2**	AACCCTGGCCACATTCAGTA	GACAGGAGTGATAGGGCCTC
**HRE 3**	GAGGCCCTATCACTCCTGTC	GGAGGAAGGATTTTGGGAGGA
**HRE 4**	CGGCTGGGTGAAGGAAATAC	GAGTTGGGGAGAACAGGGG
**HRE 5**	GAACCCCATTGCCTTTCCG	CACTCCCAGGCTCCCAAG
**HRE 6**	CTGATGCAGTCTCCTCCAGG	TCCCGAGGAACCACTTAACC

miRNA	Forward	Reverse
**mmu-miR-3085-3p mimic**	UCUGGCUGCUAUGGCCCCCUC	GGGGGCCAUAGCAGCCAGAUU
**mmu-miR-3085-3p inhibitor**	GAGGGGGCCAUAGCAGCCAGA	

miRNA reporter	Forward	Reverse
**Prkaca 3'UTR WT (Xba1, Bam H1)**	GCTCTAGAGGGTGTGCTTGTGCC	GGGATTCTCAAATATGTTTACAACAGCACA
**Prkaca 3'UTR Mutagenesis**	GGGTTGGATCGAACATAAGGAGGGCCCTAGA GTTC	GAACTCTAGGGCCCTCCTTATGTTCGATCCA ACCC

siRNA	Forward	Reverse
**si*Prkaca***	CGAGUAACUUUGACGACUA	UAGUCGUCAAAGUUACUCG
