# Supplementary material for: Adipocyte HIF2α functions as a thermostat via PKA Cα regulation in beige adipocytes
Source: Nat Commun. 2022 Jun 7;13:3268. doi: 10.1038/s41467-022-30925-0 (PMC9174489; doi:10.1038/s41467-022-30925-0)
Supplement: Supplementary file 3 — Description of Additional Supplementary Files [file 41467_2022_30925_MOESM3_ESM.pdf]

Description of Additional Supplementary Files

Supplementary Data 1.

NP score and Betweenness centrality ranks for Supplementary Fig. 6b, c

Supplementary Data 2.

Downregulated DEG in HIF2α AKO upon cold exposure
