# Supplementary material for: Adipocyte HIF2α functions as a thermostat via PKA Cα regulation in beige adipocytes
Source: Nat Commun. 2022 Jun 7;13:3268. doi: 10.1038/s41467-022-30925-0 (PMC9174489; doi:10.1038/s41467-022-30925-0)
Supplement: Supplementary file 6 — Reporting Summary [file 41467_2022_30925_MOESM6_ESM.pdf]

nature portfolio

Corresponding author(s): Jae Bum Kim

Last updated by author(s): May 17, 2022

Reporting Summary

Nature Portfolio wishes to improve the reproducibility of the work that we publish. This form provides structure for consistency and transparency in reporting. For further information on Nature Portfolio policies, see our Editorial Policies and the Editorial Policy Checklist.

Statistics

For all statistical analyses, confirm that the following items are present in the figure legend, table legend, main text, or Methods section.

n/a	Confirmed	
☐	☒	The exact sample size (*n*) for each experimental group/condition, given as a discrete number and unit of measurement
☐	☒	A statement on whether measurements were taken from distinct samples or whether the same sample was measured repeatedly
☐	☒	The statistical test(s) used AND whether they are one- or two-sided<br>*Only common tests should be described solely by name; describe more complex techniques in the Methods section.*
☒	☐	A description of all covariates tested
☐	☒	A description of any assumptions or corrections, such as tests of normality and adjustment for multiple comparisons
☐	☒	A full description of the statistical parameters including central tendency (e.g. means) or other basic estimates (e.g. regression coefficient) AND variation (e.g. standard deviation) or associated estimates of uncertainty (e.g. confidence intervals)
☐	☒	For null hypothesis testing, the test statistic (e.g. *F*, *t*, *r*) with confidence intervals, effect sizes, degrees of freedom and *P* value noted<br>*Give P values as exact values whenever suitable.*
☒	☐	For Bayesian analysis, information on the choice of priors and Markov chain Monte Carlo settings
☒	☐	For hierarchical and complex designs, identification of the appropriate level for tests and full reporting of outcomes
☒	☐	Estimates of effect sizes (e.g. Cohen's *d*, Pearson's *r*), indicating how they were calculated

Our web collection on statistics for biologists contains articles on many of the points above.

Software and code

Policy information about availability of computer code

Data collection	LAS X (CARS microscope, Leica), Axio Scan. Z1 (Zeiss), CFX 96TM (Bio-Rad), CFX Connect (Bio-Rad), Seahorse XFe96 extracellular flux analyzer (Agilent), PhenoMaster (TSE Systems)
Data analysis	ImageJ, GraphPad Prism 7, Zen, LAS X, Bio-Rad CFX Maestro, TargetScan mouse (Version 7.2), miRDB (Version 6.0), TrimGalore (version 0.4.4), the STAR aligner (version 2.6.1d), HTSeq-count (version 0.13.5), DESeq2 (version 1.30.0), STRING database (version 10.5), NetworkX package (version 2.2), clusterMaker2 (version 1.3.1), Cytoscape.

For manuscripts utilizing custom algorithms or software that are central to the research but not yet described in published literature, software must be made available to editors and reviewers. We strongly encourage code deposition in a community repository (e.g. GitHub). See the Nature Portfolio guidelines for submitting code & software for further information.

Data

Policy information about availability of data

All manuscripts must include a data availability statement. This statement should provide the following information, where applicable:

- Accession codes, unique identifiers, or web links for publicly available datasets
- A description of any restrictions on data availability
- For clinical datasets or third party data, please ensure that the statement adheres to our policy

The authors declare that the data supporting the findings of this study are available within the research article and its supplementary Information files. The accession number for RNA-seq data in this study is GSE179385.

Field-specific reporting

Please select the one below that is the best fit for your research. If you are not sure, read the appropriate sections before making your selection.

☒ Life sciences ☐ Behavioural & social sciences ☐ Ecological, evolutionary & environmental sciences

For a reference copy of the document with all sections, see nature.com/documents/nr-reporting-summary-flat.pdf

Life sciences study design

All studies must disclose on these points even when the disclosure is negative.

Sample size	Sample size for in vivo, ex vivo, and in vitro experiments was chosen based on commonly adopted standards in the field, resulting in statistically meaningful comparisons.
Data exclusions	Experiments failed for technical reasons were excluded from data analysis.
Replication	All experiments were carried out under standard and clearly defined conditions. And all attempts at replication were successful by at least one researcher.
Randomization	Randomization was used for analyzing image data. Mice from experimental and control groups were randomly selected.
Blinding	Blinding test was used for imaging experiments. And the analysis was performed in a blinded fashion whenever possible

Reporting for specific materials, systems and methods

We require information from authors about some types of materials, experimental systems and methods used in many studies. Here, indicate whether each material, system or method listed is relevant to your study. If you are not sure if a list item applies to your research, read the appropriate section before selecting a response.

Materials & experimental systems

n/a	Involved in the study
☐	☒ Antibodies
☐	☒ Eukaryotic cell lines
☒	☐ Palaeontology and archaeology
☐	☒ Animals and other organisms
☒	☐ Human research participants
☒	☐ Clinical data
☒	☐ Dual use research of concern

Methods

n/a	Involved in the study
☒	☐ ChIP-seq
☒	☐ Flow cytometry
☒	☐ MRI-based neuroimaging

Antibodies

Antibodies used	Antibodies against HIF1a (NB100-479, Novus), HIF2a (NB100-132, Novus), UCP1 (ab10983, Abcam), PKA Ca (4782S, Cell Signaling), HSL (4107S, Cell Signaling), pHSL (4139S, Cell Signaling), CREB (9197S, Cell Signaling), pCREB (9191S, Cell Signaling) pPKA substrate (9624S, Cell Signaling), pP38 (612289, BD Biosciences), P38 (Sc-7972, Santa Cruz Biotechnology), OXPHOS (ab110413, Abcam), a-tubulin (T6199, Sigma), b-actin (A5441, Sigma), and Lamin B1 (ab16048, abcam) were used.
Validation	All the antibodies were validated by manufacturer and are widely used for similar experiments by other researchers worldwide.

Eukaryotic cell lines

Policy information about cell lines

Cell line source(s)	Immortalized murine brown adipocytes (BACs), HEK293FT cells
Authentication	BAC cells were provided by Dr. Kai Ge (National Institutes of Health). HEK293FT cells were authenticated by ATCC.
Mycoplasma contamination	We used the cell lines after testing mycoplasma contamination (ROCHE 10799050001).
Commonly misidentified lines (See ICLAC register)	None

Animals and other organisms

Policy information about studies involving animals; ARRIVE guidelines recommended for reporting animal research

Laboratory animals	HIF1a AKO, HIF2a AKO and HIF1/2a DKO mice were generated by crossing Adiponectin-Cre mice with Hif1aflox/flox, Hif2aflox/flox, and Hif1aflox/flox/Hif2aflox/flox mice (C57BL/6-Hif1atm3Rsjo/J and C57BL/6-Hif2atm1Mcs/J). HIF1a BKO and HIF2a BKO mice were generated by crossing Ucp1-Cre mice with Hif1aflox/flox and Hif2aflox/flox mice.
Wild animals	The study did not involve wild animals.
Field-collected samples	The study did not involve samples collected from the field.
Ethics oversight	All experiments with mice were approved by the Seoul National University Institutional Animal Care and Use Committee (SNUIACUC).

Note that full information on the approval of the study protocol must also be provided in the manuscript.
